# Communication Disorders and Mental Health Outcomes in Children and Adolescents: A Scoping Review

**DOI:** 10.3390/healthcare13151807

**Published:** 2025-07-25

**Authors:** Lifan Xue, Yifang Gong, Shane Pill, Weifeng Han

**Affiliations:** 1 Teaching Methods Department, College of Education, Qingdao Hengxing University of Science and Technology, Qingdao 266100, China; 2 College of Education, Early Childhood Teaching, Curtin University, Perth Western 6845, Australia; 3 College of Education, Psychology and Social Work, Flinders University, Adelaide 5042, Australia

**Keywords:** communication disorders, mental health, speech–language pathology, emotional well-being, interdisciplinary care, developmental vulnerability

## Abstract

**Background/Objectives**: Communication disorders in childhood, including expressive, receptive, pragmatic, and fluency impairments, have been consistently linked to mental health challenges such as anxiety, depression, and behavioural difficulties. However, existing research remains fragmented across diagnostic categories and developmental stages. This scoping review aimed to synthesise empirical evidence on the relationship between communication disorders and mental health outcomes in children and adolescents and to identify key patterns and implications for practice and policy. **Methods**: Following the PRISMA Extension for Scoping Reviews (PRISMA-ScR) and Arksey and O’Malley’s framework, this review included empirical studies published in English between 2000 and 2024. Five databases were searched, and ten studies met the inclusion criteria. Data were charted and thematically analysed to explore associations across communication profiles and emotional–behavioural outcomes. **Results**: Four interconnected themes were identified: (1) emotional and behavioural manifestations of communication disorders; (2) social burden linked to pragmatic and expressive difficulties; (3) family and environmental stressors exacerbating child-level challenges; and (4) a lack of integrated care models addressing both communication and mental health needs. The findings highlight that communication disorders frequently co-occur with emotional difficulties, often embedded within broader social and systemic contexts. **Conclusions**: This review underscores the need for developmentally informed, culturally responsive, and interdisciplinary service models that address both communication and mental health in children. Early identification, family-centred care, and policy reforms are critical to reducing inequities and improving outcomes for this underserved population.

## 1. Introduction

Communication is central to a child’s development, serving as a foundation for learning, emotional expression, and participation in social life. From early gestures and joint attention to complex discourse, communicative competence supports not only academic growth but also identity formation and social inclusion [1]. However, for a significant number of children and adolescents, communication disorders (CDs) interrupt these developmental trajectories. These disorders—including speech sound disorder, developmental language disorder (DLD), pragmatic language impairments, and fluency disorders (i.e., stuttering)—affect approximately 7% of the paediatric population [2]. Children with CDs often experience challenges that extend beyond language, influencing peer interactions, emotional regulation, and mental well-being [3].

The intersection between communication and mental health is well-documented, though still insufficiently understood. Multiple studies suggest that children with CDs are more likely to experience internalising and externalising difficulties such as anxiety, depression, aggression, and social withdrawal (e.g., [4,5,6]). For instance, pragmatic language impairments have been linked to elevated social anxiety, while expressive language difficulties are associated with behavioural frustration and peer conflict [7,8]. Adolescents who stutter often report reduced self-esteem and increased mental distress, especially in the absence of integrated psychological and speech–language support [9]. Moreover, caregivers of children with CDs frequently report higher levels of stress, emotional burnout, and difficulty accessing coordinated services [10].

Despite a surge of empirical interest in the communication–mental health nexus, the research landscape remains fragmented. Studies vary in how they define and measure both communication and mental health constructs, leading to a lack of comparability across findings [11]. While some focus on diagnostic categories (e.g., DLD, ASD—Autism Spectrum Disorder, Attention Deficit Hyperactivity Disorder—ADHD), others examine functional traits (e.g., social withdrawal, emotional lability), and only a few employ a transdiagnostic lens. Interventions are similarly diverse, ranging from individual therapy to school-based programs, yet systematic insights into what works, for whom, and under what conditions remain limited [12]. Furthermore, populations such as culturally and linguistically diverse (CALD) children and those with co-occurring disabilities are underrepresented in this body of work [13].

Several recent studies attempted to address aspects of this relationship between communication and mental health. For example, Dillon et al. [14] explored the role of language in adolescent well-being, while Xiao et al. [15] examined associations between early communication and emotional–behavioural outcomes. However, few have adopted a broad developmental and diagnostic scope while specifically examining how communication difficulties relate to mental health trajectories in children and adolescents. To date, no study that we could find has synthesised findings across diagnostic boundaries, communication domains (expressive, receptive, pragmatic, and fluency), and mental health constructs to provide a comprehensive map of this complex intersection.

This scoping review addresses this gap. Following the PRISMA Extension for Scoping Reviews (PRISMA-ScR) and guided by Arksey and O’Malley’s [16] framework, this review synthesises peer-reviewed empirical studies on the relationship between communication disorders and mental health outcomes in children and adolescents. Specifically, it aims to address the following:

1. Identify and describe how different types of communication disorders are associated with emotional and behavioural well-being.
2. Thematise common findings and distinctions across communication profiles.
3. Highlight implications for clinical practice, educational support, and interdisciplinary policy development.

This review also considers related family-level or parental mental health outcomes, where these were reported in the context of communication disorders in children and adolescents.

## 2. Methods

The use of the PRISMA-ScR and Arksey and O’Malley’s [16] methodological framework was enhanced as suggested by Levac and colleagues [17]. Specifically, this review mapped and then synthesised peer-reviewed empirical research examining the relationship between communication disorders and mental health in children and adolescents. Given the diversity of definitions, populations, and assessment tools in the field, a scoping approach was considered most appropriate to provide a comprehensive overview of the literature for the purpose of identifying key themes for future investigation [18].

The primary research question guiding the review was: “What is the nature of the relationship between communication disorders and mental health outcomes in children and adolescents?” It explores how different types of communication difficulties (expressive, receptive, pragmatic, and fluency-related) are associated with internalising or externalising mental health symptoms, and how these associations vary across contexts, age groups, and diagnostic categories.

Studies were considered eligible for inclusion if they were peer-reviewed empirical investigations published in English between 2000 and 2024, with a primary focus on individuals under 18 years. Eligible studies needed to examine one or more communication disorders as defined by the American Speech–Language–Hearing Association (ASHA; https://www.asha.org/policy/rp1993-00208/?srsltid=AfmBOoreB78T638WUtsZrAdIzZljf4-QCY-KVpbTagcXxGOESWFx2ike, accessed on 30 May 2025) and to include at least one outcome related to mental health, such as anxiety, depression, behavioural difficulties, social withdrawal, or well-being. Quantitative, qualitative, and mixed-methods studies were eligible, provided that communication and mental health variables were both directly measured and/or meaningfully discussed. Studies were excluded if they focused solely on adult populations, described unrelated medical or developmental conditions, or were review articles, theoretical papers, editorials, or conference abstracts.

The search strategy was developed iteratively in consultation with a research librarian to ensure comprehensive coverage of the relevant literature. The final search was conducted in November 2024 across five databases: PubMed, PsycINFO, ERIC, CINAHL, and Scopus. The search terms combined key concepts related to communication disorders (e.g., “language disorder”, “speech impairment”, “pragmatic communication”, “stuttering”) and mental health outcomes (e.g., “anxiety”, “depression”, “emotional wellbeing”, “behaviour problems”) using Boolean operators. Terms were adapted for each database and included both subject headings and free-text terms. Studies were eligible for inclusion if they (1) were published in peer-reviewed journals between 2000 and 2024; (2) were written in English; (3) included participants aged under 18 years; (4) examined the relationship between communication disorders (including expressive, receptive, pragmatic, or fluency disorders) and mental health outcomes; and (5) reported empirical findings (qualitative, quantitative, or mixed-methods). Studies were excluded if they (1) were not published in English; (2) focused solely on adult populations; (3) did not report original empirical research (e.g., reviews, editorials); or (4) did not address mental health outcomes in relation to communication disorders. The full search strategy for each database is reported in Table 1.

**Figure 1:**
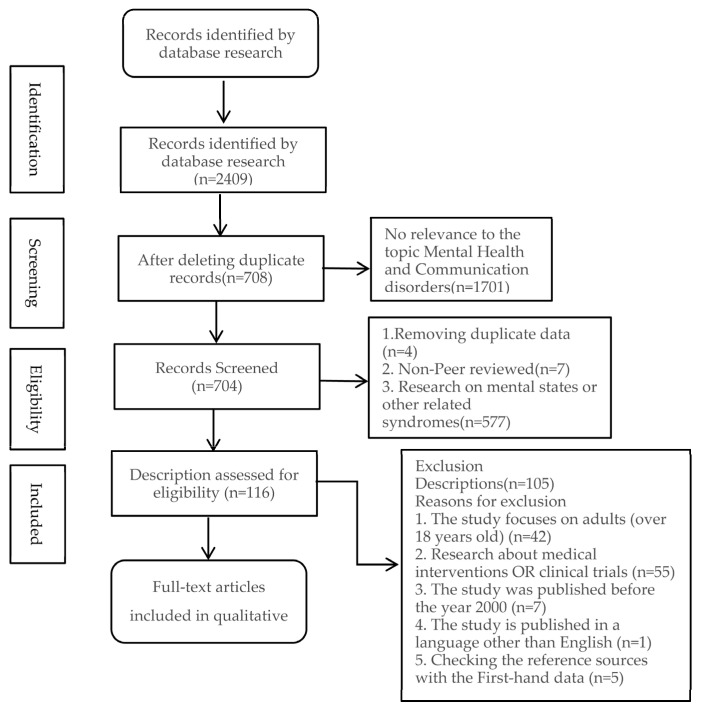
PRISMA flow diagram showing the study selection process.

All search results were exported into EndNote, and duplicates were removed before screening. Title and abstract screening were completed independently by Authors 1 and 2, who then proceeded to full-text screening for all articles deemed potentially relevant. Discrepancies were resolved through consensus discussion, with Author 3 consulted where necessary. A total of 2409 records were initially identified. After removing duplicates and screening titles and abstracts, 52 full-text articles were assessed for eligibility. Ten studies met the inclusion criteria and were included in the final synthesis. The rationale for exclusion at each stage is detailed in the diagram. This approach enhances the transparency and reproducibility of our review process. The selection process is summarised in the PRISMA flow diagram (Figure 1).

The PRISMA flow chart provides key information related to the objectives or questions of the scoping review, such as describing the results of the search strategy and selection process [19]. Key variables extracted from each study included author(s), publication year, country, study design, participant characteristics (including age and diagnostic group), type of communication disorder, mental health outcome(s) measured, assessment tools used, and key findings relevant to the review aims. The data extraction form was piloted with three studies to ensure consistency, and minor adjustments were made before proceeding with full data charting. Each study was independently charted by Author 1 and verified by Author 2 to enhance reliability. The final data extraction domains are presented in Table 2.

An inductive thematic analysis was conducted to synthesise the findings across the included studies. The process of integrating, categorising, and thematically organising data facilitates the interpretation of thematic directionality and systematic exploration within research [20]. The process focused on the nature and direction of the relationship between communication and mental health outcomes, the specific communication profiles involved (e.g., pragmatic vs. expressive), the developmental stage of participants, and the role of contextual or mediating variables such as family stress, school environment, or co-occurring conditions. Themes were developed iteratively and refined through multiple rounds of discussion among the review team. Specifically, data from included studies were independently coded by two reviewers using an inductive, iterative approach to identify recurring concepts and patterns related to communication disorders and mental health outcomes. Initial codes were compared and discussed until a consensus was reached. Themes were developed collaboratively through a process of constant comparison, with regular team meetings to review coding decisions and resolve discrepancies. Challenges included variability in study designs and reporting, which at times required negotiation and adaptation of thematic categories to best represent the underlying data. Disagreements were resolved through discussion until consensus was achieved among all reviewers. While our primary focus was on mental health outcomes among children and adolescents with communication disorders, the data extracted also contributed to synthesised findings relating to parental or family mental health where these were reported as part of the child’s experience or family context. Table 3 provides an overview of the study characteristics for the final ten included studies.

While this review does not include a formal quality appraisal of the included studies, the research team critically engaged with methodological differences and limitations in the interpretation of the findings [19]. The scope and limitations of the evidence base are discussed transparently in later sections of this paper. By systematically mapping the current evidence and identifying key patterns across communication domains and mental health outcomes, this scoping review seeks to inform clinicians, researchers, educators, and policymakers working at the interface of child development, language, and well-being.

## 3. Results

The ten studies included in this review spanned diverse populations, methodological designs, and communicative profiles, yet they shared a common concern with the interplay between language and socio-emotional functioning and their relationship to mental health outcomes in children and adolescents. Across studies, communication disorders were not only associated with a higher prevalence of emotional and behavioural difficulties but were also embedded in complex relational and systemic contexts. Through an inductive thematic analysis of the findings, four interrelated themes were identified that characterise the ways communication and mental health challenges intersect in the lives of children and adolescents. The following four themes were derived through an inductive synthesis of the data extracted from all included studies, in alignment with the review’s search and selection criteria.

### 3.1. Theme 1: Emotional and Behavioural Manifestations

Across the reviewed studies, emotional and behavioural concerns emerged as a consistent correlate of communication disorders in children and adolescents. Participants with expressive, receptive, or mixed language impairments were frequently reported to exhibit symptoms of anxiety, emotional dysregulation, irritability, and behavioural frustration. In the study by Hahn et al. [24], children with language impairment demonstrated significantly elevated internalising behaviours, including sadness, worry, and withdrawal, as compared to their typically developing peers, as rated by both parents and teachers. These emotional difficulties often co-occurred with academic underachievement and reduced classroom participation. MacEvilly et al. [12] demonstrated that group interventions can enhance emotional regulation and social communication in young children. However, they also found that language and communication barriers in young children can lead to increased internalised behaviours and decreased external interactions, potentially resulting in isolated emotional activities and heightened negative emotions.

Similar findings were echoed by Cola et al. [25], whose work on young children with language delays highlighted the presence of sustained affective symptoms, including persistent low mood and emotional lability. In some cases, these symptoms preceded formal mental health diagnoses, suggesting that emotional challenges may emerge early in the trajectory of communication difficulties. Pereira et al. [26] also reported that children with language disorders performed significantly higher on standardised behavioural assessments of emotional distress and conduct difficulties, which raised concerns about their long-term psychosocial development.

The nature of these associations appears to be both direct and mediated. For example, Maljaars et al. [21] observed that even in the absence of a formal mental health diagnosis, children with expressive and receptive difficulties displayed increased aggression and hyperactivity, possibly reflecting frustration associated with their inability to communicate effectively. This behavioural dysregulation often resulted in exclusionary responses from peers and disciplinary actions in educational settings, which further reinforced the emotional toll.

Taken together, the evidence suggests that emotional and behavioural symptoms in children with communication disorders are not peripheral but integral to their developmental profile. The findings emphasise the need for the early identification of affective difficulties within speech–language assessments and underscore the potential for mental health symptoms to escalate in the absence of coordinated intervention.

### 3.2. Theme 2: The Social Burden of Pragmatic and Expressive Difficulties

The second theme emerging from the synthesis is the significant social burden associated with pragmatic and expressive communication impairments. Children who struggled to initiate, sustain, or interpret social communication were consistently described as being at risk of social exclusion, peer victimisation, and reduced friendship quality. These difficulties often led to internalised distress and negative self-perception.

Gillott et al. [27] highlighted that children with pragmatic language deficits showed higher levels of loneliness and fewer reciprocal friendships compared to their neurotypical peers. The social withdrawal documented in their study was not simply a function of language ability, but rather, a reflection of the child’s perceived social competence and fear of negative evaluation. In the study by Charman et al. [29], children with social communication difficulties were more likely to experience peer rejection and bullying, which in turn were strongly associated with symptoms of anxiety and depression. The authors emphasised the cumulative impact of repeated negative social encounters, particularly for children lacking access to peer-mediated or school-based support. Luyster et al. [28] similarly documented that children with pragmatic language impairments often misinterpreted social cues, which exacerbated peer misunderstandings and intensified feelings of frustration and social vulnerability. In Luyster et al.’s work [28], issues such as peer misunderstandings, heightened frustration, and increased social vulnerability were identified primarily through parental reports and qualitative interviews, rather than through standardised assessment scales. These findings highlight the subjective and nuanced nature of social difficulties for children with communication disorders, as captured by informant-based accounts.

Notably, these findings suggest that the social consequences of communication disorders are not merely incidental but form a central pathway through which mental health difficulties develop and are maintained. The reviewed studies point to the need for interventions that explicitly address social communication skills in tandem with emotional coping strategies, particularly in educational environments where peer interaction is critical.

### 3.3. Theme 3: Family and Environmental Stressors

Several included studies reported on the mental health and psychological well-being of parents or carers of children with communication disorders, highlighting the interconnected nature of child and family outcomes. These findings were synthesised where they were directly linked to the experience and impact of communication disorders in children, reflecting a broader ecological perspective.

The third theme, therefore, concerns the bidirectional and compounding nature of risk factors experienced by children with communication disorders and their families. Rather than operating in isolation, communication difficulties and mental health concerns were found to interact within broader psychosocial environments, particularly in family systems where stress, limited resources, or unmet service needs were present.

Lecavalier et al. [31] suggested that parental stress levels were significantly higher among caregivers of children with both language impairments and emotional difficulties compared to those raising children with language difficulties alone. These caregivers may feel overwhelmed by the dual burden of managing communication challenges and supporting their child’s emotional well-being, especially in contexts where access to specialised care is inconsistent or fragmented.

This cyclical dynamic, whereby a child’s limited communicative repertoire exacerbated behavioural distress, which in turn intensified parental frustration, was a recurring concern in qualitative data. Family-level challenges were also influenced by structural and systemic barriers. Houghton et al. [23], for example, noted that families often struggled to navigate siloed health and education services, potentially leading to gaps in care that undermined both communication support and emotional regulation. These systemic issues were particularly pronounced for families from lower socioeconomic backgrounds or those residing in rural areas.

It is highlighted how communication and mental health challenges are embedded within broader ecological systems. Family stress, limited service access, and breakdowns in interprofessional collaboration often act as mediators that exacerbate child-level difficulties. These findings underscore the importance of adopting a family-centred, ecologically informed approach in both assessment and intervention planning.

### 3.4. Theme 4: Intervention Gaps and the Need for Integrated Care

The final theme centres on the limitations of current intervention models and the call for more integrated, interdisciplinary care. Despite strong evidence for the interdependence between communication and mental health, only a few studies reported interventions that simultaneously addressed both domains. Most speech–language therapy services described in the included studies focused narrowly on linguistic or articulatory targets, often without consideration of the child’s emotional state or psychological support needs.

While emotional distress is often identified in children with communication disorders, referral pathways to mental health services were inconsistent and poorly coordinated [29]. Caregivers may also be unaware that speech and emotional concerns could be addressed jointly, resulting in fragmented care and delayed psychological support [25]. A notable concern raised across studies was the lack of school-based or community programs that support both communication competence and emotional well-being. While there is potential for classroom-level interventions to promote social–emotional learning among children with communication difficulties [25], few programs have been formally trialled or evaluated in this population. This reflects a broader gap in the literature and practice: the absence of dual-focus models that bridge developmental, educational, and psychological disciplines.

The findings point to a pressing need for interdisciplinary models of care that position speech–language therapists, psychologists, educators, and families as collaborative agents in a child’s development. Such models could support timely screening, reduce service duplication, and ensure that emotional well-being is treated as integral, rather than incidental, to communicative functioning.

### 3.5. Intersecting Pathways of Communication and Mental Health: The Conceptual Framework

**Figure 2:**
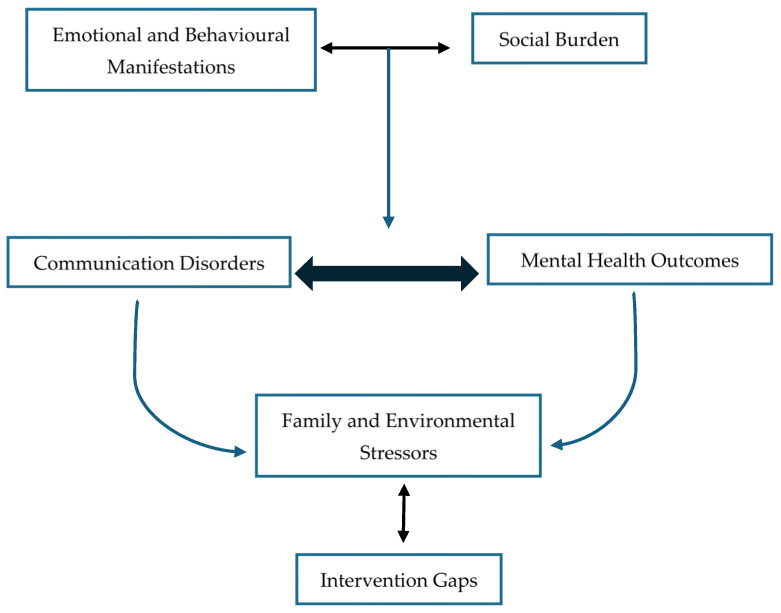
Conceptual framework illustrating the interconnected pathways between communication disorders, mental health outcomes, social environments, and systemic support. Communication disorders and mental health outcomes are depicted as mutually influencing and co-occurring, with bidirectional arrows indicating that difficulties in one domain may exacerbate challenges in the other. Social burdens (such as peer rejection, stigma, and social withdrawal) and emotional/behavioural manifestations (such as anxiety, frustration, and conduct problems) are shown as mediating factors that both result from and contribute to this interplay. Systemic support and barriers (e.g., access to integrated care, educational accommodations) influence all domains, highlighting the importance of contextual and structural factors in shaping outcomes.

The four themes coalesce into a conceptual framework that maps the dynamic and reciprocal relationships between communication disorders and mental health outcomes in children and adolescents. As illustrated in Figure 2, the framework proposes that communication disorders and mental health outcomes in children and adolescents are interrelated and frequently co-occur, with reciprocal influences over time. For example, children with communication difficulties may experience social exclusion or misunderstanding, which increases their risk of emotional and behavioural problems. Conversely, mental health challenges can compound communication difficulties, creating a cycle of vulnerability. The social environment (e.g., family support, peer relationships, school climate) and broader systemic supports or barriers (such as availability of interdisciplinary care or inclusive education policies) interact with both domains, either mitigating or exacerbating difficulties. This model highlights the complex, multi-layered pathways through which communication disorders and mental health outcomes are intertwined, emphasising the need for comprehensive, context-sensitive intervention strategies.

At the foundation of the model lies Theme 1, where communication difficulties are directly associated with internalising and externalising symptoms such as anxiety, mood disturbance, and behavioural dysregulation. These difficulties, in turn, restrict children’s ability to engage meaningfully in everyday settings, leading to cumulative emotional strain. Building on this foundation, Theme 2 highlights how pragmatic and expressive impairments carry a particular burden in the social domain. Children with these challenges frequently encounter peer rejection, miscommunication, and reduced social reciprocity, which not only intensify emotional difficulties but also contribute to the development of a negative self-concept. Theme 3 extends this interaction to the family and environmental level. The distress experienced by children is often mirrored by or compounded within their caregiving systems. Parents and guardians experience emotional fatigue, limited access to integrated care, and systemic navigation challenges. These stressors create feedback loops that reinforce communication and behavioural challenges over time. Finally, Theme 4 underscores the absence of coordinated care models that recognise and respond to these intersecting needs. Without integrated interventions, children and families are left to navigate fragmented services, delaying support and amplifying the developmental impact of both communication and mental health challenges.

Collectively, this framework illustrates that communication disorders do not operate in isolation. Rather, they participate in a network of reinforcing factors that impact mental health, functioning, and quality of life. Interventions targeting only one dimension of risk overlook the broader ecosystem of needs experienced by these children and their families.

## 4. Discussion

This review examined the complex relationship between communication disorders and mental health difficulties in children and adolescents, synthesising insights from current research and contextualising them within a broader body of literature. The findings underscore the multifaceted and interdependent nature of these challenges, which manifest not only at the individual level but also across social, familial, and systemic domains. In this section, we interpret the key themes identified, relate them to existing research, and consider their implications for future directions in research, practice, and policy.

### 4.1. Revisiting the Communication–Mental Health Nexus

The findings of this review confirm and extend the well-documented yet complex relationship between communication disorders and mental health challenges in childhood and adolescence. While the co-occurrence of these two domains is acknowledged in the literature, the present synthesis demonstrates that emotional and behavioural difficulties are not merely coexisting conditions, but rather, are often intertwined consequences of communicative breakdown, social disconnection, and systemic limitations. This interaction was evident across all types of communication disorders examined, including expressive and receptive language difficulties, pragmatic language impairments, and fluency disorders.

The results reinforce that children with communication disorders are at elevated risk of both internalising and externalising symptoms. Across the included studies, anxiety, withdrawal, low mood, and behavioural frustration were frequently observed. Importantly, these mental health symptoms were not only secondary to communication difficulties; in some cases, they appeared concurrently or even prior to formal diagnosis, suggesting a mutually reinforcing developmental pathway. This aligns with evidence (e.g., [11]) indicating that communication deficits and mental health concerns in early childhood may share neurodevelopmental origins, with cascading effects over time. This argues for a transdiagnostic perspective, where cognitive–affective vulnerabilities contribute to both domains simultaneously [5].

Moreover, the review adds to emerging work that challenges the assumption that communication impairment alone accounts for poor social or emotional outcomes. It shows that the child’s social environment, peer responses, and access to support are equally important in shaping outcomes. The current synthesis contributes to this literature by mapping how these contextual influences interact with core communication profiles to heighten or mitigate psychological vulnerability.

Overall, this review supports a reframing of communication disorders, not merely as isolated developmental delays but as potential drivers of broader emotional, behavioural, and psychosocial difficulties. This reconceptualisation carries significant implications for clinical assessment, which must attend to signs of emotional distress alongside speech–language profiles, and for early intervention approaches that are responsive to both communicative and emotional needs from the outset [32].

### 4.2. Social and Family Systems as Mediators of Risk

This review highlights that the relationship between communication disorders and mental health difficulties is not shaped solely by individual impairments but by the broader social and familial contexts in which children develop. Across the included studies, peer rejection, poor friendship quality, caregiver stress, and family coping difficulties consistently emerged as influential mediators that intensified the emotional burden associated with communicative challenges.

Children with pragmatic language impairments or expressive delays were particularly vulnerable to social exclusion and peer conflict. These findings align with broader research, such as Clarke [4] and Channell and Bosley [5], who documented that difficulties in understanding or producing socially appropriate language can contribute to breakdowns in peer interaction, which in turn impact self-concept and emotional regulation. Further, adolescents with language difficulties are often found to internalise peer rejection as a personal failure, leading to increased anxiety and reduced help-seeking behaviours [6].

Family dynamics also played a critical role in shaping child outcomes. For example, caregivers with co-occurring communication and emotional difficulties with their children might show higher levels of stress, exhaustion, and frustration [31]. These responses often stemmed from persistent communicative breakdowns, difficulty interpreting the child’s needs, and a lack of access to mental health support services. Leitão et al. [33], for example, indicated that caregivers of children with communication disorders experience significantly higher levels of stress than caregivers of typically developing children, with stress compounded by service system delays and inconsistent professional support.

The corpus suggests that the family environment can serve as both a buffer and an amplifier. In emotionally responsive households with coordinated access to intervention, children may experience improved outcomes despite language difficulties. However, in contexts marked by financial hardship, cultural or linguistic mismatch, or limited professional support, these same challenges may escalate. It needs to be emphasised that the accumulation of social and familial stressors, rather than the severity of the communication disorder alone, is often a stronger predictor of negative psychosocial outcomes [34].

This reinforces the value of ecological models of development (such as Bronfenbrenner’s ecological systems theory; see [35]), which account for the interactions between the children, their immediate relationships, and broader societal structures. Interventions that focus only on the child, without considering the family’s coping capacity and the social affordances around them, are unlikely to achieve sustainable improvements. Therefore, a shift toward family-centred and socially informed practice is essential in bridging the gap between communication development and emotional well-being.

### 4.3. Systemic Barriers and Gaps in Interdisciplinary Practice

Despite growing recognition of the link between communication disorders and mental health among the studies reviewed, the findings still reveal a persistent lack of integration between speech–language pathology and psychological services. Across the included studies, participants and caregivers reported fragmented service delivery, poorly coordinated referral pathways, and limited access to practitioners trained to support both communication and emotional needs. This siloed model of care is at odds with the complex, bidirectional nature of the challenges experienced by children with co-occurring difficulties.

One of the most striking patterns emerging from the review is the absence of routine emotional well-being screening within speech–language therapy settings. Although speech–language pathologists often reported concerns about their paediatric clients’ affective state or behavioural regulation, formal mental health referral processes were inconsistently applied, and only a few multidisciplinary care plans were implemented. Doody et al. [36] identified several obstacles to effective care planning and implementation. Privacy and confidentiality were highlighted as primary concerns, alongside the crucial role of trust in interactions with health professionals, which significantly impacts the success of care plans. This echoes the claims (e.g., [37]) that identify considerable variation in Australian interprofessional practices involving speech–language pathologists and other allied health professionals, largely due to jurisdictional differences in service mandates and funding models.

Structural limitations significantly hinder effective care, as highlighted by Langbecker et al. [38] and Cocquyt et al. [10]. These include long waitlists, insufficient school-based resources, and poor inter-sector communication. Compounding these issues, children often receive initial speech–language services but lack timely access to psychological support unless their symptoms reach a crisis level. This reactive approach neglects the crucial developmental and preventative needs of this population. Meanwhile, while existing programs, e.g., the PICP program, assess language skills, specifically receptive speech and passive coping styles, in children receiving speech and language services, their broader effectiveness needs further exploration. For example, Pereira et al. [26] observed improved language skills in preschoolers with pragmatic disabilities using this program, but more research is necessary to determine its efficacy across various neurodevelopmental disorders.

There also appears in the corpus a systemic underinvestment in training that prepares professionals to work collaboratively across disciplines. While some school-based programs aim to foster multidisciplinary teams, many practitioners report limited confidence in managing overlapping issues beyond their core expertise (e.g., [12]). As a result, opportunities for early, coordinated, and contextualised support are often missed.

The findings of this review suggest an urgent need to reconceptualise service delivery models for children with communication disorders who present with or are at risk of mental health concerns. Integrated assessment protocols, joint case planning, and co-delivered interventions should become standard practice rather than exceptional arrangements. Investment in training, policy alignment, and interdisciplinary research is also essential to build evidence-based models that reflect the developmental interdependence of communication and mental health.

### 4.4. Underrepresented Populations and Equity Concerns

Another critical observation arising from this review is the marked underrepresentation of equity-focused perspectives in the research on communication disorders and mental health. Despite the well-established evidence that social determinants of health, such as socioeconomic disadvantage, cultural and linguistic diversity, and geographic location, profoundly shape access to care and developmental outcomes, few current studies meaningfully address these variables. This omission is particularly concerning given the disproportionate burden of communication difficulties and mental health challenges experienced by children from minoritised communities.

Socioeconomic status (SES) also intersects with both communication and mental health needs. Children from low-SES families are more likely to experience both language delay and elevated emotional distress yet face higher thresholds to access services. Systemic constraints (including out-of-pocket costs, reduced service availability in rural areas, and limited school-based support) significantly reduce opportunities for early intervention [39]. These inequities not only widen developmental gaps but also entrench disadvantages across educational and health domains.

The lack of equity-oriented research designs in the corpus of research also limits the generalisability and translational value of the findings. A number of studies are still conducted with predominantly white, English-speaking, urban samples, often with limited contextual information about family background, linguistic diversity, or community resources. Without intentional inclusion of diverse populations, both the evidence base and the interventions derived from it risk reinforcing existing disparities.

Addressing these gaps, in the context of linguistic diversity and community resource support, requires an explicit shift toward equity-informed inquiry and practice. This includes recruiting diverse participant samples [40], developing culturally and linguistically responsive assessment tools [41], and embedding equity considerations in service design and delivery [42]. It also requires a policy-level commitment to resourcing services in underserved communities and supporting the cultural competence of the workforce. Without these steps, children from minoritised backgrounds will continue to be underrepresented in research and underserved in practice, despite bearing a disproportionate share of the burden.

### 4.5. Translating Findings into Research, Practice, and Policy

The findings of this review point to a pressing need for more cohesive, responsive, and inclusive approaches across research, clinical practice, and policy settings. While the association between communication disorders and mental health difficulties is increasingly recognised, current responses remain fragmented and inadequate to address the complexity of children’s lived experiences.

From a research perspective, future studies must move beyond documenting co-occurrence and instead examine the mechanisms by which communication and mental health challenges influence one another across developmental stages. Longitudinal and mixed-method designs are especially needed to track trajectories over time and capture nuanced family and community influences. Importantly, there is a clear imperative for studies to prioritise the inclusion of children from underrepresented populations, particularly those from CALD, Indigenous, low-SES, and rural or remote communities, as the absence of such representation not only limits generalisability but also exacerbates inequities by failing to reflect the realities of those most in need of support.

In clinical practice, the findings of this review underscore the value of embedding emotional and behavioural monitoring within routine speech–language assessments. Practitioners should be supported to identify signs of distress, refer appropriately, and collaborate across disciplinary lines. This includes shared goal setting with psychologists, joint case management, and co-delivered school-based interventions. Studies included in this review have shown the potential of such interdisciplinary models but also highlight the need for clearer pathways and more consistent training in interprofessional collaboration (e.g., [43,44,45]). Strengthening reflective practice, trauma-informed care, and culturally responsive communication strategies will also be essential to enhancing service relevance and reach. Furthermore, to advance integrated models of care, it is important to draw on principles from implementation science and health services research. These fields offer frameworks for translating evidence into practice, highlighting factors such as stakeholder engagement, fidelity, sustainability, and the importance of context in the adoption of interdisciplinary interventions [46,47]. Incorporating these perspectives can help ensure that recommendations for integrated, culturally responsive, and family-centred care are both feasible and sustainable in real-world settings.

Additionally, at the policy level, there is an urgent need for investment in integrated care models that are developmentally and culturally informed. These must include coordinated funding across education, health, and community sectors, particularly for early intervention and school-based programs. In this sense, multi-agency planning structures and shared outcome frameworks can facilitate more efficient and family-centred service delivery [48]. Policy also plays a critical role in mandating equity-focused research funding, supporting the training of a diverse workforce, and embedding co-design principles that elevate the voices of children and families in shaping services.

Ultimately, this review reinforces the need to move beyond siloed, diagnosis-driven systems towards holistic, intersectional approaches that address the interwoven nature of communication, emotional well-being, family dynamics, and systemic structures. Children and young people will be best served when research, practice, and policy work in concert to acknowledge and respond to this complexity.

## 5. Strengths, Limitations, and Future Directions

This scoping review makes a timely and substantive contribution to understanding the intersection between communication disorders and mental health in childhood and adolescence. By synthesising thematic insights from a diverse set of studies and situating them within a broader field of emerging research, this review offers a multidimensional account of how communicative challenges and emotional well-being interact across developmental and social contexts.

A key strength of this review lies in its focus on the interwoven, rather than parallel, nature of communication and mental health difficulties. The identification of four cross-cutting themes, namely, emotional–behavioural manifestations, social burden, familial stress, and systemic barriers, provides a coherent framework that advances both theoretical understanding and clinical application. The review also applies an ecologically grounded lens, drawing attention to underexplored variables such as cultural identity, socioeconomic position, and interprofessional practice. This approach supports a more inclusive and contextually relevant account of children’s lived experiences.

Nonetheless, several limitations must be acknowledged. A key limitation of this scoping review is the inclusion of only ten studies, which restricts the breadth and generalisability of our findings. This narrow evidence base partly reflects our decision to limit the search to English-language, peer-reviewed empirical publications. While this approach ensured consistency in methodological quality and feasibility of data extraction, it may have excluded relevant studies published in other languages or within the grey literature, particularly from underrepresented or non-English-speaking contexts. The exclusion of these sources limits the ability to fully capture the global and cultural diversity of experiences at the intersection of communication disorders and mental health. We recommend that future reviews consider expanding the inclusion criteria to encompass non-English language publications and grey literature. Such an approach would offer a more comprehensive synthesis and help address the underrepresentation of certain populations and settings in the existing literature.

Another key limitation of the present review is the heterogeneity in how mental health outcomes were measured and reported across included studies. Assessment tools ranged from standardised diagnostic measures to qualitative descriptions and proxy indicators, resulting in inconsistencies that complicated direct comparison and synthesis. This methodological variability limits the strength of any generalisations or causal inferences that can be drawn. Future reviews would benefit from stratifying findings based on the methodological rigour of included studies, which may help clarify developmental trajectories and relationships between communication disorders and mental health outcomes.

While the available evidence did not allow for a systematic comparison of mental health outcomes across specific subtypes of communication disorders, this remains an important area for future research. More granular investigation is needed to understand how distinct forms of expressive, receptive, pragmatic, or fluency difficulties may confer differential risk for particular emotional or behavioural challenges, with implications for tailored clinical assessment and intervention. Future research, therefore, should prioritise longitudinal and mixed-method designs to examine causal pathways and resilience factors. Specifically, this research needs to address the disparity in developmental support for disadvantaged groups, who often lack access to language-rich environments and adequate social resources. Greater inclusion of culturally and linguistically diverse participants, as well as co-designed studies involving caregivers, practitioners, and young people, will be critical to advancing equity and impact. There is also a need to develop and evaluate integrated service models that bring together speech–language and mental health support, particularly in early childhood and school settings. Such approaches hold promise not only for enhancing developmental outcomes but also for preventing the escalation of difficulties into adolescence and beyond.

## 6. Conclusions

This scoping review aimed to synthesise empirical evidence on the relationship between communication disorders and mental health outcomes among children and adolescents, while considering family-level or parental mental health outcomes where these were reported in the context of childhood communication disorders. Our findings address these aims through four interconnected themes that map the multidimensional and systemic nature of these associations. Overall, it underscores the urgent need to reframe how communication disorders and mental health are understood and addressed in children and adolescents. These challenges rarely occur in isolation; rather, they are deeply intertwined, shaped by social relationships, family dynamics, and systemic service barriers. Thematic synthesis revealed that communication difficulties can both mask and magnify emotional distress, often going unrecognised in siloed systems of care. Families face compounding pressures, while many children remain underserved due to fragmented support, cultural mismatch, or inequitable access. In summary, this scoping review advances our understanding of how communication disorders and mental health outcomes intersect in childhood and adolescence, while also identifying the influence of family and systemic factors. By explicitly addressing our stated aims, this review highlights both established patterns and significant gaps that warrant further research and targeted intervention.

Given the limited number and heterogeneity of the included studies, the conclusions of this scoping review should be interpreted as preliminary and hypothesis-generating rather than definitive. While the findings indicate key areas for attention in practice and policy, further research is needed to substantiate and refine these recommendations, particularly with more diverse and robust data. To move forward, therefore, research must centre diverse voices and examine developmental pathways through longitudinal and contextually grounded designs. Practice must shift toward integrated, family-centred models. Policies must fund and mandate interprofessional, culturally responsive services that reach beyond diagnostic categories. Addressing the co-occurrence of communication and mental health needs is not only clinically necessary but an equity imperative. Supporting these children early and holistically has the potential to shift trajectories, reduce the long-term burden, and promote resilience across systems.

## Figures and Tables

**Table 1:** Search terms related to communication disorders and mental health outcomes in the ASD population.

Population	Qualifier	Exclusion
communication disorder/(language disorder* OR speech disorder*)/language impairment/articulation disorder/phonological disorder/speech sound disorder/(dysarthria* OR stuttering*)/fluency disorder/language delay/specific language impairment/developmental language disorder/expressive language disorder/(aphasia* OR dysphasia*)/cognitive-communication disorder/pragmatic language disorder	“children” OR “kid*” OR “adolescents” OR “youth” OR “pediatric” OR “childhood” OR “school-aged children” OR “early childhood”	Other physical or other disorders other than communication disorders.
Mental health/(Anxiety* OR Social Anxiety Disorder)/Autism Spectrum Disorder/ Depression	“children” OR “kid*” OR “adolescents” OR “youth” OR “pediatric” OR “childhood” OR “school-aged children” OR “early childhood”	Populations unrelated to the research topic (e.g., Mental state: Refers to a person’s mental state at a specific moment or in a short period of time, which may be temporary and fluctuating.)

**Table 2:** Data items extracted.

Study Type	Domain	Data Items
Intervention Study	Study details Participant characteristics Barriers	Author, year, title, content, number of participants Language background, settings of intervention
Descriptive	Study details Participant Demographics Outcomes/Contributions Rehabilitation Practices	Intervention practices, keywords Author, year, title, topic relevance, publication type Use of interpreters, adapting rehabilitation Recruitment, research, policy and advocacy

**Table 3:** Full list of included texts.

Citation	Study Design	Participant Characteristics	Communication Disorder(s) Assessed	Mental Health/Social Outcome(s) Measured	Key Finding(s)
[21]	Experimental	36 children with Autistic Disorder and Intellectual Disability (AD + ID); 26 with ID only; 34 typically developing (TD). Age range: 3.3–11.3 years (AD + ID).	Receptive and expressive language.	Social development (impacted by language); problem behaviours associated with receptive language impairment.	Children with AD + ID had higher expressive than receptive language scores, a pattern opposite to that of the comparison groups. In the AD + ID group, joint attention and symbol understanding were most strongly related to language abilities.
[22]	Experimental (Brief Report; Re-analysis of data)	20 boys with high-functioning autism; 23 TD boys. Age range approx. 8–11 years.	Inner speech.	Social symptoms of autism (as related to cognitive profile).	Children with autism who had a nonverbal > verbal (NV > V) skills profile showed significant inner speech impairment, unlike other groups. This suggests a link between cognitive profile and inner speech deficits.
[23]	Experimental (Intervention Study)	6 children with autism (experimental group); 6 matched controls with autism. Age range: 47–78 months.	Child-initiated social communication, including social orienting and gestural communication.	Social engagement, duration of social interactions.	Following a 40 h intensive Son-Rise Program, children in the treatment group showed a significant increase in spontaneous social orienting, gestural communication, and the duration of social interactions compared to controls.
[24]	Longitudinal (Descriptive)	28 children with Fragile X Syndrome (FXS). Ages assessed at a “toddler period” (24–36 months) and a “child period” (59–68 months).	Joint Engagement (JE), receptive and expressive language.	Autism Spectrum Disorder (ASD) symptomatology.	Higher levels of joint engagement in toddlers with FXS were concurrently associated with more advanced expressive language and negatively related to autism symptomatology. Early JE also predicted better expressive and receptive language skills later in childhood.
[25]	Experimental	48 children and teens with Autism Spectrum Condition (ASC); 50 neurotypical (NT) peers. Age range: 7–17 years.	Conversational adaptation (measured by “talkativeness”/word count) across different social contexts.	Social communication competence and behavioural adaptation.	NT participants adapted their talkativeness to match their partner (spoke less with a bored partner), whereas ASC participants did not, maintaining a consistent level of talkativeness across both contexts.
[26]	Non-randomised controlled trial (Intervention Study)	20 preschool-age children with ASD or Developmental Language Disorder (DLD); 11 in intervention group, 9 in control. Age range: 3; 6–6; 11 years.	Pragmatic impairments (e.g., joint attention, turn-taking, communicative initiative).	Socialisation and mental health difficulties (as a consequence of pragmatic impairments).	A pragmatic intervention program (PICP) led to significant improvements in parent- and teacher-rated pragmatic goals and general language ability for children in the intervention group compared to the control group.
[27]	Descriptive (Comparative)	15 children with Specific Language Impairment (SLI); 15 with high-functioning autism; 15 TD children. Age range: 8–12 years.	Phonologic-syntactic language impairments.	Theory of Mind (ToM) ability (social cognition).	Both the SLI and autism groups gave fewer correct mental state answers on a ToM task than TD peers. The autism group made more inappropriate mental state errors, while the SLI group did not differ from controls in this respect.
[28]	Descriptive	164 toddlers with ASD. Age range: 18–33 months.	Receptive and expressive language.	Social–cognitive skills (joint attention, imitation, gesture use) as correlates of language.	Gesture use and non-verbal cognitive ability were the most significant concurrent predictors for both receptive and expressive language. Response to joint attention also predicted receptive language, while imitation predicted expressive language.
[29]	Descriptive (Comparative)	62 children with Language Impairment (LI); 42 with ASD, all in mainstream schools. Age range: 5–13 years.	Language Impairment (LI).	Emotional and behavioural problems (emotional, conduct, hyperactivity, peer problems) via the Strengths and Difficulties Questionnaire (SDQ).	Both the LI and ASD groups showed similarly elevated levels of emotional, conduct, and hyperactivity problems. The ASD group had higher peer problems and lower prosocial behaviour scores.
[30]	Longitudinal	516 infants with high (HL) or low likelihood (LL) for autism; 81 were later diagnosed with autism (HL-ASD).	Early social communication skills (e.g., emotion/eye gaze, gestures, sounds, words, understanding).	Language development as a downstream outcome of social communication skills.	Infants later diagnosed with autism showed widespread reductions in social communication skills at 12 months. Unlike other groups, the association between social communication and later language did not emerge in the ASD group until 24 months of age.

## Data Availability

No new data were created.

## Abbreviations

ADHD	Attention-deficit hyperactivity disorder
ASD	Autism Spectrum Disorder
ASHA	American Speech–Language–Hearing Association
CALD	Culturally and linguistically diverse
CDs	Communication disorders
DLD	Developmental language disorder
SES	Socioeconomic status
